# A Practical Treatise on Materia Medica and Therapeutics

**Published:** 1889-04

**Authors:** 


A PRACTICAL TREATISE ON MATERIA MEDICA AND THERAPEUTICS. By Robert S. Bartholow, M. A., M. D., LL. D. Sixth edition. New York: D. Appleton & Co. 1888. pp. xxiv, 802.

